# Impact of preoperative cannabis use on outcomes following surgical intervention for gastroparesis

**DOI:** 10.1007/s00464-025-12558-8

**Published:** 2026-01-20

**Authors:** Sven E. Eriksson, Naveed Chaudhry, Margaret E. Gardner, Tara Zarrineh, Ping Zheng, Shahin Ayazi

**Affiliations:** 1 Chevalier Jackson Esophageal Research Center, Western Pennsylvania Hospital, Allegheny Health Network, Pittsburgh, PA USA; 2 Foregut Division, Surgical Institute, Allegheny Health Network, Pittsburgh, PA USA; 3 Department of Medicine, Baylor Scott & White Health, Temple, TX USA; 4 Department of Surgery, Drexel University, Philadelphia, PA USA

**Keywords:** Gastroparesis, Cannabis, Pyloroplasty, G-POEM, Gastric electrical stimulation

## Abstract

**Introduction:**

Cannabis use has been associated with delayed gastric emptying and may worsen symptoms in patients with gastroparesis. However, its impact on postoperative outcomes after surgical treatment remains unclear. This study aimed to evaluate postoperative outcomes among patients with and without preoperative cannabis use undergoing surgical intervention for gastroparesis.

**Methods:**

A retrospective cohort study was conducted using the TriNetX federated electronic medical record database. Adults with gastroparesis who underwent pyloric drainage surgery or gastric stimulator placement were stratified by cannabis use (ICD-10 F12) prior to surgery. Outcomes were assessed over the first 90 days and up to 5 years postoperatively. Statistical comparisons included risk analysis, Kaplan–Meier survival, and number of instances.

**Results:**

The study cohort consisted of 1,572 patients, including 108 (6.9%) with documented preoperative cannabis use. Patients with cannabis use were younger (39.8 vs 44.6 years, p = 0.002) and had higher rates of diabetes (56.5% vs 45.1%, p = 0.033), GERD (77.7% vs 65.1%, p = 0.014), nausea/vomiting (85.2% vs 59.4%, p < 0.001), and abdominal pain (73.1% vs 50.2%, p < 0.001). Within 90 days of surgery, cannabis users experienced higher rates of reintervention (9.3% vs 1.2%, p < 0.001) and inpatient admissions (32.4% vs 23.7%, p = 0.042), but fewer clinic visits (37.0% vs 54.5%, p < 0.001). By 5 years, cannabis users demonstrated persistently greater inpatient utilization with higher hospital admission rates (59.3% vs 41.0%, p < 0.0001) and shorter time to first admission (log-rank p < 0.001). Outpatient engagement was significantly lower, with fewer patients attending follow-up (54.6% vs 76.2%, p < 0.001), fewer total clinic encounters (8.1 vs 12.3, p = 0.045), and longer time to first clinic visit (log-rank p < 0.001). Rates of gastrectomy (0.0% vs 0.7%, p = 0.541) and late reintervention (12.0% vs 11.3%, p = 0.808) were similar between groups.

**Discussion:**

Preoperative cannabis use is associated with a greater symptom burden, increased early reintervention, reduced outpatient engagement, and greater long-term healthcare utilization after surgical intervention for gastroparesis. These patients may represent a more symptomatic population and may benefit from closer follow-up to reduce readmissions.

**Graphical Abstract:**

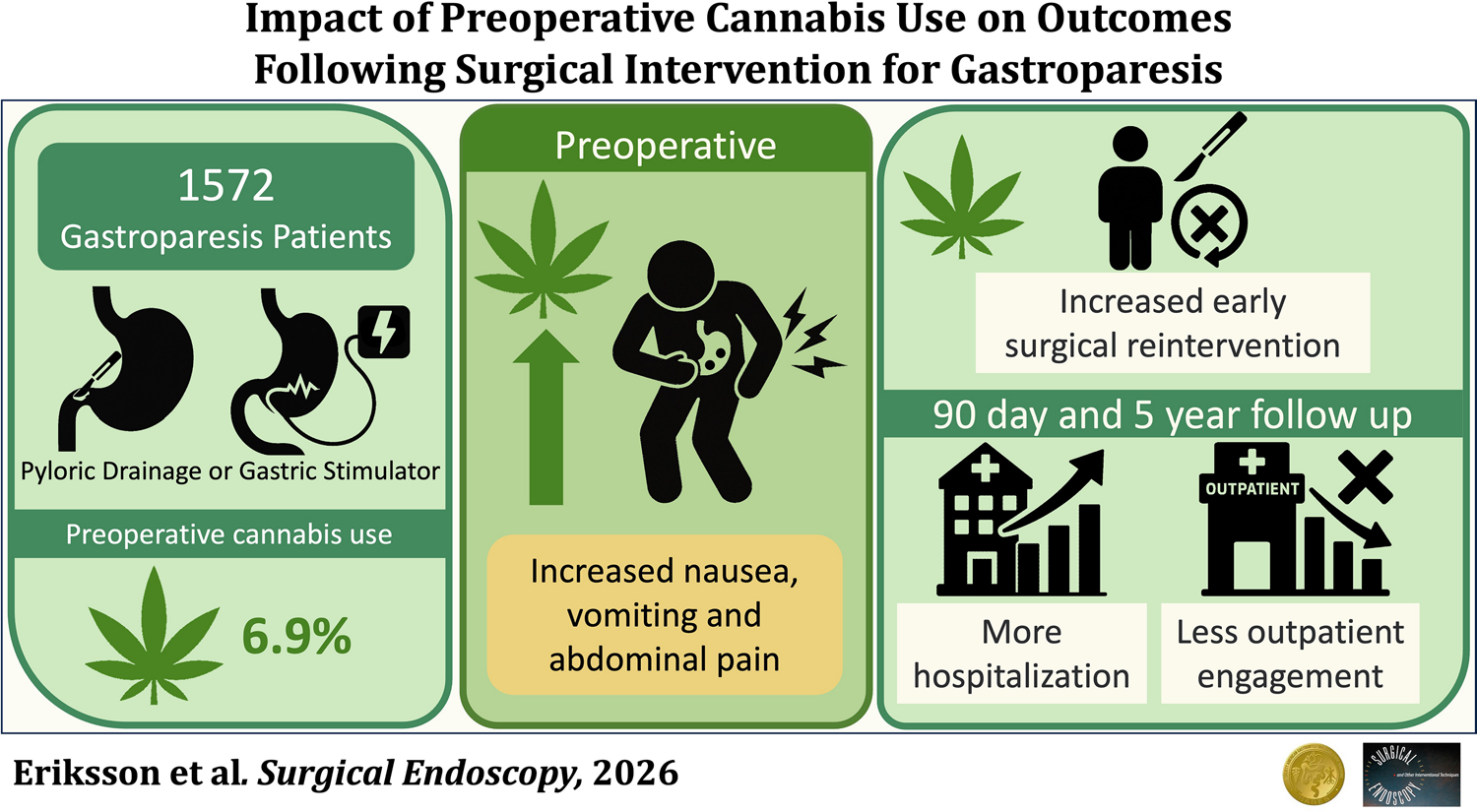

Gastroparesis is a chronic gastric motility disorder characterized by upper gastrointestinal symptoms with objective evidence of delayed gastric emptying in the absence of mechanical obstruction [1–3]. Evaluation with endoscopy or imaging is necessary to exclude mechanical obstruction before establishing the diagnosis. Common symptoms include postprandial fullness, nausea, vomiting, upper abdominal pain, early satiety, and bloating. [1–3]. The condition substantially impairs quality of life, is frequently refractory to medical therapy, and contributes to significant healthcare utilization and cost [4]. For patients with severe, medically refractory disease, surgical options such as pyloric drainage, by pyloroplasty or gastric per-oral endoscopic myotomy (G-POEM), or gastric electrical stimulation may be considered [3, 5, 6].

Cannabis use has risen sharply in recent years, driven by both expanding legalization and patient interest in its perceived therapeutic benefits for gastrointestinal symptoms [7, 8]. Cannabinoids, particularly Δ⁹-tetrahydrocannabinol (THC), have well-characterized effects on gastrointestinal physiology, including suppression of antral contractility, delayed gastric emptying, and modulation of visceral pain and central nausea pathways [9, 10]. Although some patients with chronic gastrointestinal disorders report symptomatic relief with cannabis, these effects may paradoxically worsen gastric stasis. Furthermore, prolonged exposure has been linked to cannabinoid hyperemesis syndrome [11, 12].

Cannabis use also carries distinct perioperative implications. Chronic exposure has been linked to altered anesthetic requirements, cardiopulmonary variability, and higher rates of postoperative nausea, vomiting, and pain [12–14]. These physiologic effects raise concern that cannabis use may influence recovery and healthcare utilization after surgery. However, despite the growing prevalence of cannabis use in the general surgical population, its impact on outcomes following surgical management of gastroparesis remains poorly defined.

The impact of cannabis on gastric physiology and its perioperative effects suggest that its use may influence outcomes following surgical treatment for gastroparesis. The present study aims to characterize the population of cannabis users seeking surgical intervention for gastroparesis and to evaluate the association between preoperative cannabis use and postoperative outcomes among patients undergoing pyloric drainage surgery or gastric electrical stimulation. Outcomes of interest included healthcare utilization and need for additional procedures within 90 days and up to five years after surgery.

## Methods

### Study design and patient cohort

This was a retrospective cohort study assessing the impact of cannabis use on surgical outcomes in patients who underwent pyloric drainage surgery or gastric electrical stimulator placement for the management of gastroparesis. This study utilized patient data from TriNetX (Cambridge, MA, USA), a nationwide federated research network that compiles fully de-identified electronic health record data from more than 100 participating healthcare organizations across the United States, representing tens of millions of patients. The network integrates inpatient and outpatient information, including demographics, International Classification of Diseases, Tenth Revision, Clinical Modification (ICD-10-CM) codes, Current Procedural Terminology (CPT) codes, and medication records obtained from both pharmacy and clinical data. Contributing organizations include academic and community hospitals as well as outpatient centers, encompassing both insured and uninsured patient populations. Data within the TriNetX network are continuously updated through automated extraction from institutional electronic health records and undergo standardized quality control procedures to ensure consistency and accuracy across sites. All information is de-identified in compliance with the Health Insurance Portability and Accountability Act (HIPAA) Privacy Rule, with verification by an independent expert. Only aggregate data and statistical summaries are available to investigators, and patient counts fewer than ten are concealed to preserve confidentiality. Preoperative cannabis use served as the primary exposure variable, and postoperative clinical outcomes were evaluated as the primary endpoints.

Adult patients aged 18–89 years with a diagnosis of gastroparesis (ICD-10 K31.84) who underwent pyloric drainage surgery or gastric electrical stimulator placement were included. Surgical procedures were identified using CPT codes for pyloroplasty (43,800) and laparoscopic implantation or replacement of gastric neurostimulator electrodes (43,647). Patients were stratified into two cohorts based on the presence or absence of a cannabis-related diagnosis (ICD-10 F12) recorded prior to surgery. The study was designed to evaluate the association between preoperative cannabis use and postoperative outcomes across all surgical modalities, rather than to compare outcomes between different surgical procedures.

### Outcomes and follow*-*up

Postoperative outcomes were evaluated over the five years following the index surgery for gastroparesis. Outcomes of interest included the need for additional or revisional pyloroplasty or gastric stimulator surgery, need for gastrectomy, prescriptions for gastroparesis-related medications, and postoperative healthcare utilization. Healthcare utilization outcomes included hospital admissions and observation encounters (CPT 1013659, 1,013,648), emergency department visits (CPT 1013712), and outpatient clinic visits (CPT 1013638). These encounters were captured to quantify postoperative healthcare demand across both inpatient and outpatient settings. Medication outcomes focused on prescriptions for prokinetic and antiemetic agents commonly used in the management of gastroparesis, including metoclopramide, erythromycin, ondansetron, prochlorperazine, simethicone, and dronabinol, identified using RxNorm terminology. Follow-up was measured from the date of surgery to the last recorded encounter or event within the network. Outcomes were analyzed across two predefined intervals: a long-term period extending up to five years after surgery and an early postoperative period encompassing the first 90 days.

### Statistical analysis

Patients were divided into two cohorts based on preoperative cannabis use: those with a cannabis-related diagnosis (ICD-10 F12) and those without. Postoperative outcomes were compared between cohorts using risk analysis, Kaplan–Meier survival analysis, and event-frequency analyses. For binary outcomes (revisional surgery, gastrectomy, hospital admission, emergency department visit, outpatient clinic visit, and medication), between-group comparisons used the Pearson chi-square test, with Fisher’s exact test applied when expected cell counts were < 10; absolute and relative risk, odds ratio, risk difference, 95% confidence intervals, and associated test statistics and p values were reported. Time-to-event outcomes were evaluated using Kaplan–Meier methods, with survival curves compared by the log-rank test and hazard ratios presented with 95% confidence intervals. Event frequency (number of admissions, clinic encounters, emergency department visits, and medication prescriptions per patient) was compared using independent-samples t tests, with mean, standard deviation, and p values reported. Analyses were performed over an early postoperative period, comprising the first 90 postoperative days, and a long-term period of up to five years. All analyses were conducted within the TriNetX Analytics platform on de-identified, aggregate-level data.

## Results

### Study population

The final study population consisted of 1,572 patients who underwent surgical intervention for gastroparesis. The mean age was 44 ± 16 years, 77% were female, and the mean BMI was 27 ± 7 kg/m^2^. Gastroparesis was associated with diabetes mellitus in 47% of patients. Common presenting symptoms included nausea or vomiting in 61%, abdominal pain in 51%, and bloating or distension in 14%. Among these patients, 108 (6.9%) had a documented cannabis-related diagnosis prior to surgery, while 1,464 (93.1%) had no record of cannabis use.

Demographics and preoperative clinical characteristics are compared between cannabis use groups in Table 1. Patients who used cannabis were significantly younger and there was a trend toward more males (p = 0.054). Cannabis use in gastroparesis was also associated with diabetes and GERD. Patients who used cannabis also more frequently complained of nausea/vomiting and abdominal pain. Neither BMI nor bloating/distension was associated with cannabis use. Patients who used cannabis were also more likely to use tobacco (45.3% vs 13.3%, p < 0.0001) and abuse alcohol (10.2% vs 1.5%, p < 0.0001). But opiate use was similar between groups (6.5% vs 3.8%, p = 0.196). At the time of surgery, patients who used cannabis were more likely to be taking metoclopramide (76.9% vs 51.0%, p < 0.0001) and domperidone (9.3% vs 2.7%, p = 0.0002). There was no difference in erythromycin use (22.2% vs 20.3%, p = 0.630).

**Table 1:** Comparison of demographics and associated medical history between patients with vs without preoperative cannabis use

Characteristic	Preop Cannabis Use (N = 108)	No Cannabis Use (N = 1,489)	p-value
Age, mean (SD)	39.8 (12)	44.6 (16)	0.002
Female sex, %	69.4%	77.5%	0.054
BMI, mean (SD)	26.4 (7.7)	27.2 (6.7)	0.259
Diabetes, %	56.5%	45.1%	0.033
GERD, %	77.7%	65.1%	0.014
Nausea/Vomiting, %	85.2%	59.4%	< 0.001
Abdominal pain, %	73.1%	50.2%	< 0.001
Bloating/Distension, %	12.0%	14.0%	0.519

### Early postoperative outcomes

Early postoperative outcomes within 90 days are summarized in Table 2. Patients with a preoperative cannabis diagnosis demonstrated reduced outpatient engagement, with fewer cannabis users presenting to clinic, and the total number of clinic encounters in the cannabis cohort was also lower compared with non-users.

**Table 2:** Comparison between cannabis use groups of early postoperative healthcare utilization within 90 days of surgery

Outcome	Cannabis Use (n = 108)	No Cannabis (n = 1,464)	*p* value
Patients with a hospital admission, %	32.4%	23.7%	0.042
Mean number of admissions ± SD	0.74 ± 1.81	1.11 ± 7.18	0.594
Patients with an ED visit, %	21.3%	16.3%	0.180
Mean number of ED visits ± SD	0.62 ± 1.77	0.37 ± 1.46	0.087
Patients with a clinic visit, %	37.0%	54.5%	< 0.001
Mean number of clinic visits ± SD	1.06 ± 1.97	1.33 ± 1.93	0.150

However, emergency department utilization was higher among cannabis users, with more patients presenting for unscheduled evaluation. Hospital admissions were also more frequent in the cannabis cohort, though the mean number of inpatient encounters was similar between groups. Kaplan–Meier analysis also demonstrated a trend toward earlier hospitalization in the cannabis cohort (*p* = 0.069, log-rank test).

Revisional procedures for gastroparesis were infrequent overall but occurred more often among cannabis users (9.3% vs 1.2%, p < 0.001).

### Five-year outcomes

Patients were followed for up to five years, with a mean ± SD follow-up of 2.9 ± 1.8 years. Mean follow-up was 2.5 ± 1.7 years in the cannabis cohort and 2.9 ± 1.8 years in the non-cannabis cohort (p = 0.030). Postoperative healthcare utilization outcomes are compared between groups in Table 3.

**Table 3:** Comparison 5-year postoperative healthcare utilization between cannabis use groups

Outcome	Cannabis Use (n = 108)	No Cannabis (n = 1,464)	p value
Patients with a hospital admission, %	59.3%	41.0%	< 0.0001
Mean number of admissions ± SD	5.11 ± 17.71	4.76 ± 29.10	0.902
Patients with an ED visit, %	39.8%	35.3%	0.346
Mean number of ED visits ± SD	4.47 ± 11.90	2.76 ± 9.17	0.068
Patients with a Clinic visit, %	54.6%	76.2%	< 0.0001
Mean number of clinic visits ± SD	8.08 ± 17.59	12.32 ± 21.38	0.045

**Fig. 1:**
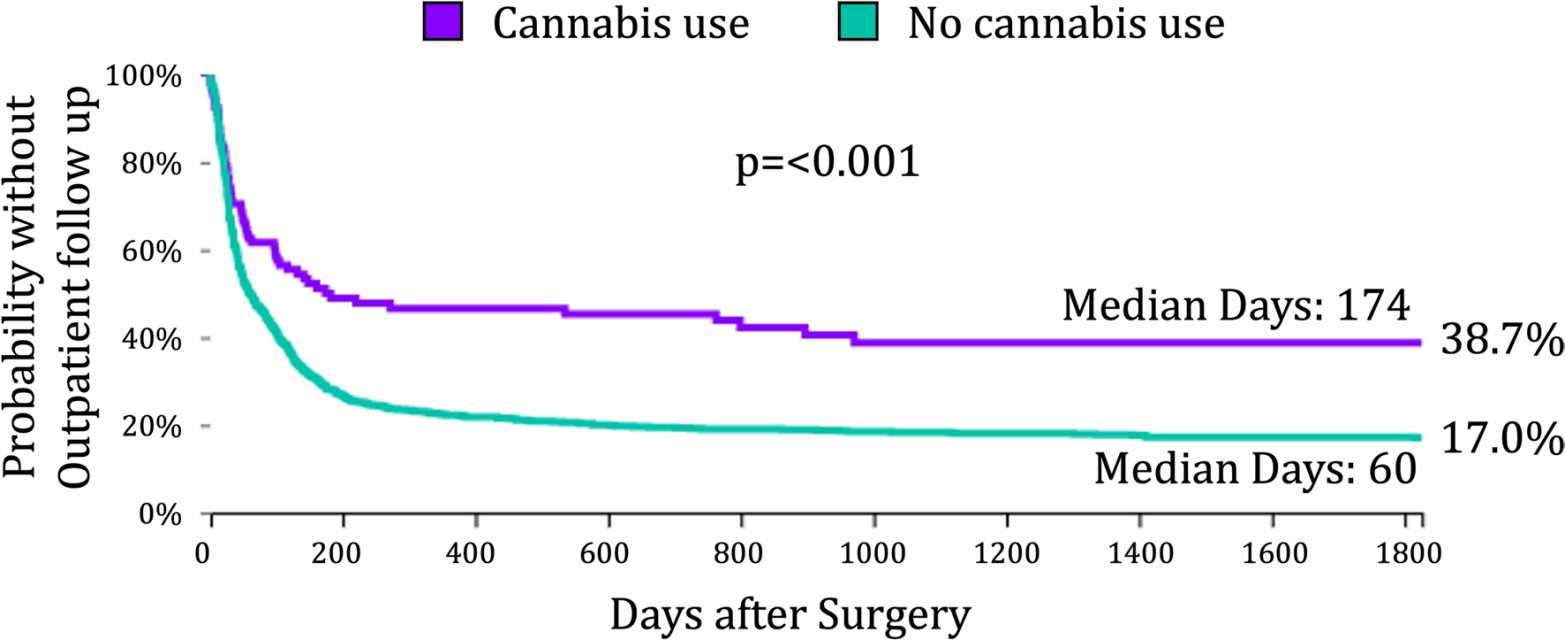
Time to first outpatient clinic visit following surgical intervention for gastroparesis. Kaplan–Meier analysis demonstrating the probability of remaining without an outpatient clinic encounter after surgery in patients with and without a cannabis-related diagnosis. Cannabis users had a significantly longer median time to first clinic encounter (174 vs 60 days, p < 0.0001) and a lower cumulative probability of follow-up throughout the postoperative period (p < 0.0001, log-rank test). At five years, 38.7% of cannabis users remained without follow-up compared with 17.0% of non-users

**Fig. 2:**
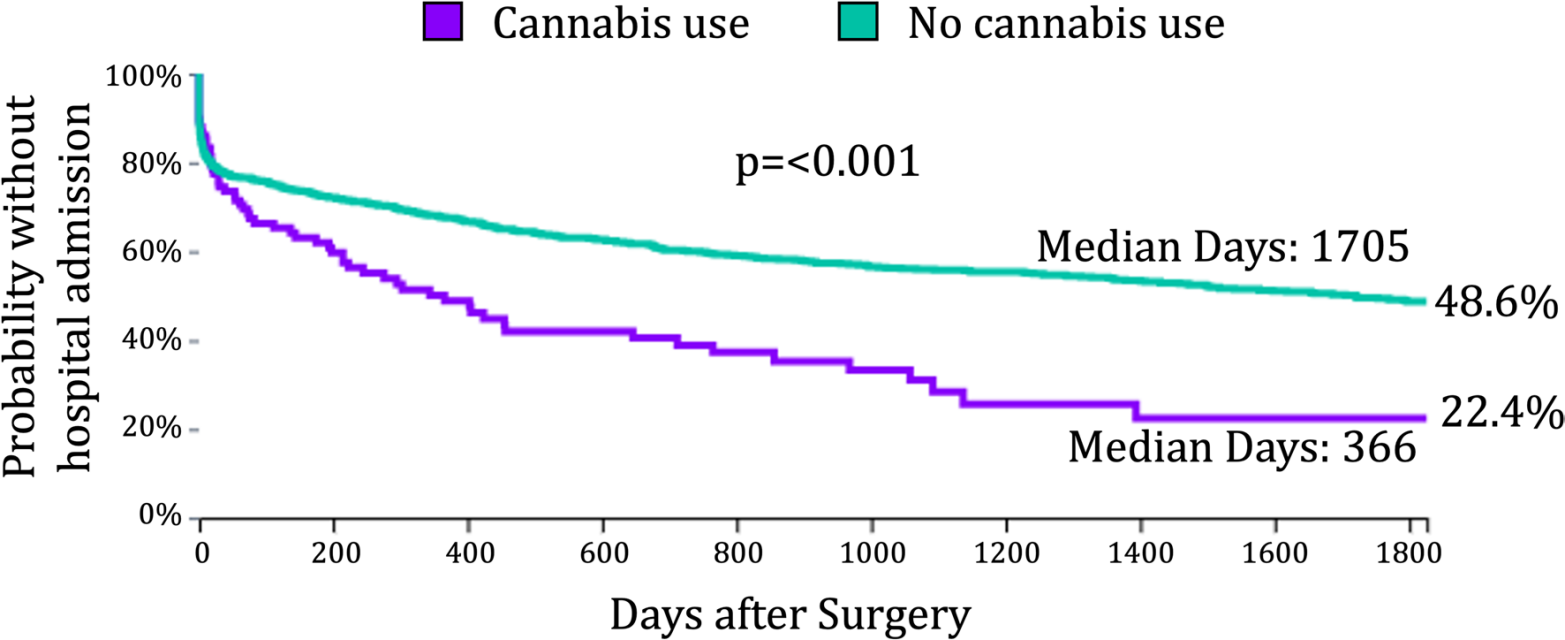
Time to first hospital admission following surgical intervention for gastroparesis. Kaplan–Meier analysis demonstrating the probability of remaining free from hospital admission after surgery in patients with and without a cannabis-related diagnosis. Cannabis users had a significantly shorter median time to first hospital admission (366 vs 1,705 days, p < 0.0001) and greater cumulative probability of hospitalization over follow-up (p < 0.0001, log-rank test). At five years, 48.6% of cannabis users remained admission-free compared with 22.4% of non-users

Patients who used cannabis were significantly less likely to attend an outpatient clinic visit and had fewer total clinic visits than non-users. Time to first clinic encounter was also significantly longer in the cannabis cohort (Fig. 1). Conversely, cannabis users had a higher number of emergency department visits and were more likely to require hospital admission (p < 0.0001). Kaplan–Meier analysis demonstrated an earlier and greater probability of hospitalization in the cannabis cohort (Fig. 2).

Need for revisional gastroparesis surgery was similar between groups (12.0% vs 11.3%, p = 0.808). Gastrectomy was rare overall and comparable between groups (0.0% vs 0.7%, p = 0.541).

## Discussion

Over the past two decades, expanding legalization and changing public attitudes toward cannabis have led to a marked rise in its use across the United States. National estimates report up to 15.3% of the adult population used cannabis in the last year, and both the frequency and proportion of daily users have nearly doubled during this period. Among patients with gastroparesis cannabis use has similarly increased, with national estimates suggesting that roughly 1 in 10 affected individuals report current use [15–18]. Prior studies have linked cannabis use to greater symptom severity and altered healthcare utilization in the medically managed gastroparesis population, but the impact of cannabis on surgical outcomes has not been well characterized [15, 16, 19]. In this large, retrospective cohort study, we found that patients who used cannabis prior to surgical intervention for gastroparesis had a substantially higher symptom burden, particularly nausea, vomiting, abdominal pain symptoms, and were more likely to present with comorbid diabetes and GERD. Postoperatively, cannabis users experienced significantly higher rates of early surgical reintervention, greater short- and long-term inpatient utilization, and persistently lower outpatient follow-up. Despite these differences, long-term rates of reintervention and gastrectomy did not differ significantly, suggesting that preoperative cannabis use may be a surrogate for higher symptom burden and healthcare barriers, rather than diminished surgical efficacy.

In our surgical cohort, 7.25% of patients had documented preoperative cannabis use. This proportion is slightly lower than that reported in broader gastroparesis populations, where multicenter cohort data show 11.7% of patients reporting current use and national inpatient data demonstrate a 9% prevalence during hospitalizations for gastroparesis [18, 20]. The lower prevalence in our study may reflect undercoding in the preoperative setting, differences in patient selection for surgery, or patient reluctance to disclose use before an elective operation. Regardless of the cause, patients with gastroparesis who use cannabis represent a distinct subset of surgical candidates who should be identified preoperatively for accurate preoperative risk assessment and perioperative planning.

Cannabis users in our cohort exhibited a greater preoperative symptom burden, most prominently nausea, vomiting, and abdominal pain and greater rates of diabetes and GERD, Similar trends have been observed in prior studies of the impact of cannabis use in medically managed gastroparesis. One such study found that patients with gastroparesis who use cannabis reported significantly higher vomiting and upper abdominal pain scores compared to non-users (2.7 vs 2.1 and 3.5 vs 2.9, respectively). Additionally, cannabis users demonstrated greater use of antiemetics such as promethazine and cannabinoid-based therapies such as dronabinol [20]. Also consistent with our findings, large national database study demonstrated that 45% of gastroparetic cannabis users had diabetes compared to 30% of gastroparetic non-users (p < 0.001) [19]. These consisted data demonstrate that cannabis users constitute a more symptomatic and metabolically complex subset of the gastroparesis population which should be taken into account when they present seeking intervention.

The greater baseline symptom severity among cannabis users likely contributes to their higher postoperative healthcare utilization. Within 90 days of surgery, these patients had significantly higher rates of reintervention and hospital admission yet participated less in outpatient follow-up. This imbalance persisted over long-term follow-up, with cannabis users continuing to demonstrate higher inpatient use and lower outpatient engagement. Similar trends have been described in non-surgical populations. In a 1:1 propensity-matched cohort study of 41,347 patients with gastroparesis who did vs did not use cannabis, they found that cannabis use was associated with significantly higher odds of emergency department visits (OR 1.73) and hospitalizations (OR 1.44) [19]. Similarly, a National Readmission Database study identified cannabis use as an independent predictor of 30-day readmission following hospitalization for gastroparesis (OR 1.7) [21]. Our results extend these observations to the surgical population, suggesting that cannabis use may be a marker for a high-utilization phenotype even after definitive interventions such as pyloric drainage or gastric stimulator placement.

Interestingly, despite a higher rate of inpatient admissions, there is some evidence to suggest that when cannabis users are admitted, they have better inpatient outcomes. A National Inpatient Sample database study of nearly 1.5 million gastroparesis patients found that cannabis users hospitalized for gastroparesis had shorter lengths of stay (3.7 vs 5.5 days, p < 0.001) and lower in-hospital mortality (0.1% vs 0.9%, p < 0.001) compared to non-users [22]. However, these findings may reflect confounding from younger age, lower comorbidity burden, and lower socioeconomic status demonstrated in their study (all p < 0.001). Similarly, our cannabis-using patients were significantly younger, which may contribute to faster recovery and shorter hospitalizations. Future studies should clarify whether the higher admission rates observed among cannabis users stem from greater symptom burden or from socioeconomic barriers that limit outpatient access and drive reliance on emergency care.

The higher healthcare utilization observed among cannabis users in our study may reflect both incomplete symptom control and differences in how cannabis is used across patient populations. Many patients turn to cannabis to relieve refractory nausea, vomiting, or abdominal pain, yet its real-world effectiveness remains uncertain. In our surgical cohort, cannabis users presented with greater preoperative symptom burden and required more inpatient care postoperatively, suggesting that cannabis often provides inadequate or transient relief. Small therapeutic studies have reported symptomatic improvement with cannabinoid agents; for example, an open-label trial of 24 patients treated with marijuana, dronabinol, or both demonstrated significant reductions in GCSI nausea/vomiting, fullness/satiety, and bloating/distention scores [23]. However, these findings are limited by small sample size, lack of controls, and non-standardized dosing. The disparity between controlled studies and population-level outcomes likely reflects differences between medically supervised use and self-directed or recreational consumption, as well as the tendency for more symptomatic patients to self-medicate.

Beyond inadequate symptom control, physiologic and anesthetic impact from cannabis itself may also contribute to the higher early reintervention and hospitalization rates. Prior studies on non-gastroparetic cannabis users have demonstrated increased perioperative risk in this population. One large multicenter study reported that cannabis use disorder was associated with broadly increased in perioperative morbidity and mortality after any major elective non-cardiac surgery (OR 1.19) [24]. These results suggest that cannabis can affect perioperative outcomes independent of gastroparesis. Another study demonstrated altered anesthetic requirements, heightened postoperative pain, and more frequent nausea and vomiting among cannabis users [13]. Together, these findings reinforce that cannabis use may predispose surgical patients to greater postoperative care needs, which may contribute to early increased utilization and need for additional intervention. Conversely, a propensity-matched analysis of colectomy patients, found no significant association between preoperative cannabis use disorder and composite morbidity, anastomotic leak, or length of stay [25]. This difference likely reflects variation in disease pathology, surgical complexity, and postoperative physiology between colectomy and gastroparesis operations. In our cohort, the combination of greater preoperative symptom burden, cannabis-related physiologic effects, and distinct perioperative responses likely contributed to the higher rates of early postoperative utilization observed among cannabis users.

Prior research may have characterized cannabis use patterns, symptom severity, and healthcare utilization in gastroparesis broadly, yet there remains a critical gap in understanding its impact in the surgical setting. No published studies to date have examined outcomes following pyloric drainage or gastric stimulator placement in the context of cannabis use. Most available literature focuses on medical management or general hospitalizations, without attention to the unique perioperative and postoperative considerations of surgical gastroparesis care. Our findings therefore extend the existing body of knowledge by identifying cannabis use as a potential risk factor for increased early postoperative morbidity and long-term inpatient utilization in this specific population. Recognizing this association has important implications for preoperative counseling, perioperative planning, and postoperative follow-up strategies, and highlights the need for prospective studies to clarify causality and guide targeted interventions.

This is the first study to specifically evaluate the impact of cannabis use on postoperative outcomes in patients undergoing surgery for gastroparesis using a large, multi-institutional, real-world dataset. The findings are strengthened by both short- and long-term follow-up and the inclusion of surgical procedures relevant to advanced gastroparesis care. However, several limitations must be acknowledged, including the retrospective design and the limited granularity inherent to large administrative databases. Cannabis use was identified through ICD-10 coding, which likely underestimates true prevalence and does not capture usage patterns, dosage, or cannabinoid composition. Insurance status and other access-to-care variables were not available in the database and therefore could not be assessed as potential confounders. Unmeasured factors such as socioeconomic status, mental health comorbidities, and barriers to care may also influence both cannabis use and postoperative outcomes. Despite these limitations, the large and diverse study population provides broad generalizability, and the consistency of findings across multiple time points supports the robustness and clinical relevance of the observed associations. Procedure-specific subgroup comparisons were not performed, as the goal of this study was to evaluate cannabis use as the primary exposure across the surgical gastroparesis population rather than to compare surgical techniques.

Cannabis use appears to identify patients with greater clinical complexity rather than directly impairing surgical outcomes. Recognizing this association provides an opportunity to improve perioperative planning and patient engagement. Incorporating cannabis use into preoperative assessment can help tailor counseling, set realistic expectations, and coordinate resources for postoperative support and outpatient continuity. For gastroenterologists, awareness of this relationship may prompt earlier multidisciplinary involvement for patients whose symptom burden or healthcare utilization suggests elevated risk.

Future research should move beyond association to mechanism. Prospective studies must quantify cannabis exposure in terms of frequency, route, and composition, and evaluate its physiologic effects on gastric motility, pain response, and anesthetic requirements. Equally important is understanding patient behavior: why individuals with gastroparesis turn to cannabis, how it shapes their engagement with care, and whether barriers to outpatient access drive reliance on acute services. Clarifying these factors will enable development of targeted perioperative protocols and refine risk stratification for the growing subset of surgical patients who use cannabis.

## Conclusion

Cannabis use exerts well-defined effects on gastric physiology that may uniquely influence outcomes in gastroparesis. In this large multi-institutional cohort, cannabis use identified a distinct subset of surgical patients with greater symptom burden, higher rates of diabetes and GERD, increased early postoperative interventions, and more frequent hospital admissions, accompanied by lower outpatient engagement over five years. These findings underscore the importance of incorporating cannabis use into preoperative risk assessment and perioperative planning for patients undergoing surgery for medically refractory gastroparesis.
